# The Direct and Moderating Effect of Food Insecurity on Obesity—A Cross‐Sectional Study

**DOI:** 10.1002/fsn3.72193

**Published:** 2026-07-30

**Authors:** Burak Mete, Hatice Merve Sadıkoğlu, Fatma İrem Dağlı, Şeyma Nur Kutay Güler, Hakan Demirhindi, Ferdi Tanır

**Affiliations:** ^1^ Department of Public Health, Faculty of Medicine Çukurova University Adana Turkey

**Keywords:** energy‐dense, food security, obesity, poverty, protein

## Abstract

Although a substantial body of evidence indicates that food insecurity is positively associated with obesity, the underlying mechanisms of this relationship remain unclear. This cross‐sectional study, conducted on 859 individuals, aimed to examine the association between food insecurity and obesity. Food insecurity was assessed using the Household Food Insecurity Access Scale (HFIAS), which was developed by the Food and Nutrition Technical Assistance (FANTA) project under the United States Agency for International Development (USAID). Factors influencing food insecurity and the role of food insecurity in obesity were evaluated. The mean age of the participants was 34.70 ± 13.84 years (range: 18–85). The prevalence of obesity was 4.5% in the mildly food‐insecure group, 12% in the moderately food‐insecure group, and 11.3% in the severely food‐insecure group (*p* = 0.013). The odds of obesity were 2.075 times higher among those experiencing severe food insecurity. The odds of severe food insecurity were 1.59 times higher in men and 2.50 times higher in rural residents, but 2.8 times lower in households with an income above the poverty line (Odds Ratio [OR] = 0.356) and 1.87 times lower in those receiving social support (OR = 0.532). Individuals with moderate and severe food insecurity were found to consume less protein‐rich foods and more grain‐based (carbohydrate) and snack products daily (*p* < 0.001). Food insecurity was found to be a significant moderator in the relationship between protein intake and body mass index, with a stronger association observed at lower levels of food insecurity and a weaker association at higher levels. Food insecurity was associated with higher obesity prevalence, and this association was moderated by dietary patterns, specifically lower intake of protein‐rich foods and higher consumption of cereals and snack foods. Providing social support to at‐risk groups may help reduce obesity risk.

## Graphical abstract

This cross‐sectional study conducted in Türkiye investigated the direct and moderating effects of food insecurity on obesity prevalence. The findings reveal that food insecurity is associated with a graded increase in obesity rates: mild food insecurity raised obesity prevalence by 4.5%, moderate insecurity by 12%, and severe insecurity by 11.3%. Notably, severe food insecurity more than doubled the risk of obesity, an effect largely mediated by a dietary shift characterized by reduced protein intake and increased consumption of energy‐dense, nutrient‐poor foods such as grains and snacks. Importantly, food insecurity was found to moderate the relationship between protein intake and body mass index (BMI), suggesting that the protective effect of dietary protein on weight management diminishes under conditions of food insecurity. These results underscore the need for integrated public health strategies that address both food access and diet quality to mitigate obesity risk in vulnerable populations.

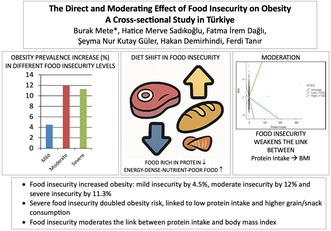

## 1 Introduction

Food insecurity refers to the limited or uncertain availability of nutritionally adequate and safe foods, or the limited or uncertain ability to acquire acceptable foods in socially acceptable ways. According to *The State of Food Security and Nutrition in the World 2025* (SOFI 2025) report by the Food and Agriculture Organization (FAO) et al. (2025), 2.33 billion people globally experienced regular difficulties in accessing sufficient food, and 828 million people faced severe food insecurity. The globalization of food trade, a growing global population, climate change, and rapidly evolving food systems all influence food security (World Health Organization [WHO] 2024). According to the results of the 2022 Global Food Security Index (GFSI) report, global food security continues to deteriorate. The report emphasized the continued increase in food prices since 2019 (largely due to the COVID‐19 pandemic) and the resulting difficulties in accessing healthy, nutritious, and sufficient food (The Economist Group 2022). The shocks experienced between 2020 and 2022 have increased the fragility of the global food system and threaten food security. Shocks such as COVID‐19, conflicts, extreme weather events and rising costs are exacerbating systemic problems and weakening the resilience of the system (World Economic Forum 2022). Interstate conflicts are on the rise, and the number of shocks related to climate change, such as droughts and floods, has increased significantly in the 21st century (FAO 2019; Palik et al. 2020). Climate‐related food system shocks, which used to occur on average every 12 years, now occur approximately every 2.5 years (Kray et al. 2022). The combination of these shocks weakens the overall resilience of the global food system, negatively affecting its ability to prepare for and withstand disruptions in order to ensure an acceptable and accessible food supply (FAO 2019). According to the results of the 2022 GFSI report, global food security continues to deteriorate, and Turkey ranks 49th among 113 countries (The Economist Group 2022). Turkey has a significant competitive advantage due to its rich biodiversity, agriculture‐based industry, natural structure, climatic characteristics, geographical location, and ranking among the top 10 producers of nearly 55 products (FAO et al. 2025). However, since 2020, Turkey has experienced major shocks such as the COVID‐19 pandemic‐related cost inflation, extreme weather events linked to climate change (frost, hail, drought, etc.), regional conflicts (the Syrian war, the Russian‐Ukrainian war), and the Kahramanmaraş earthquakes, resulting in a period of inflation, which is expected to continue (Disaster and Emergency Management Authority [AFAD] 2023; FAO 2023; Turkish Statistical Institute [TÜİK] 2023; World Bank 2023). The final outcome of all these catastrophic events has been an increase in food prices. Food prices in Turkey increased by 9.04% in 2020, 18.11% in 2021, 55.61% in 2022, 71% in 2023, 69.71% in 2024, and 41.76% in 2025 (Central Bank of the Republic of Turkey [CBRT] 2025).

The relationship between food insecurity and obesity, known as the “food insecurity–obesity paradox,” has been explained by the “insurance hypothesis” (Bateson and Pepper 2023) and the preference for energy‐dense, low‐cost foods among lower‐income individuals (Drewnowski and Specter 2004; Darmon and Drewnowski 2015). Limited access to nutritious and safe food is becoming an increasingly pressing issue (United Nations World Food Programme [WFP] 2025). Although a positive association is generally reported, findings remain conflicting and are influenced by factors such as gender, marital status, stress, and food assistance programs (Franklin et al. 2012). A recent meta‐analysis reported that food insecurity was associated with a 1.42‐fold increased risk of obesity (Moosavian et al. 2026).

## 2 Materials and Methods

### 2.1 Research Type and Ethics

This cross‐sectional study was conducted at primary care settings in 2025 by the Department of Public Health in the Faculty of Medicine at Çukurova University. A total of four rural family health centres and two healthy life centres located in the Adana city centre, Turkey, were included in the study. The study population consisted of individuals aged 18 years or older who presented to the mentioned centres for routine primary healthcare and preventive health services. Ethical approval was obtained from the Non‐Interventional Research Ethics Committee of the Faculty of Medicine, Çukurova University (Date: 07.02.2025, Decision No: 152).

### 2.2 Determination of Sample Size and Selection of Participants

The minimum sample size was calculated using a power of 99%, a type I error (α) of 0.05, and an effect size (*f*) of 0.20, yielding a required sample of 752 participants. Participants were recruited using a convenience sampling method during their visit to primary care institutions. Concerned that selecting a non‐probability sampling method could lead to a decrease in the statistical representativeness and power of the study, the calculated sample size was increased by approximately 15%, reaching 859 individuals.

### 2.3 Measuring Instruments

After the individuals were informed about the aim of the study, written informed consents were obtained. They were administered a face‐to‐face questionnaire consisting of two sections: The first section included sociodemographic characteristics, anthropometric measurements, and questions on factors affecting nutritional status. The second section included the Household Food Insecurity Access Scale (HFIAS), the Food Frequency Questionnaire (FFQ), and the Malnutrition Universal Screening Tool (MUST). The assessment of poverty was performed by using OECD equivalence scales.

#### 2.3.1 Anthropometric Measurements

The participants' height (cm) and weight (kg) were measured. Body mass index (BMI) was calculated using the formula: BMI = weight (kg)/height^2^ (m^2^). According to the WHO classification, participants were categorized as follows: underweight (< 18.5 kg/m^2^), normal weight (18.5–24.9 kg/m^2^), overweight (25.0–29.9 kg/m^2^), and obese (≥ 30.0 kg/m^2^).

#### 2.3.2 Assessment of Poverty

Participants were asked to report their total monthly household income as an open‐ended question. Per capita income was calculated using the modified OECD equivalence scale. This scale, developed by Hagenaars et al. (1994) is widely used by both official institutions and academic studies in Turkey (Chanfreau and Burchardt 2008). In this scale, the household head is assigned a value of 1.0, each additional adult 0.5, and each child 0.3. Individuals aged 14 or above are considered adults. The household number and distribution of the family members were obtained based on the declaration of the participants (Oztornacı and Demirdogen 2015). Per capita daily income was calculated in United States Dollars (USD) based on the exchange rate at the time of data analysis. According to the World Bank income classification for 2024–2025, Turkey is considered an upper‐middle‐income country. The poverty threshold for this group is USD 6.85 per day. Participants' income levels were categorized according to this threshold (Kofi et al. 2024; Metreau et al. 2024).

##### 2.3.2.1 Assessment of Food Expenditure

The proportion of income spent on food was calculated by dividing the total monthly household food expenditure by the total monthly household income and multiplying by 100 to obtain a percentage. Participants were asked to report their total monthly household income and total monthly food expenditure as open‐ended questions. Food expenditure included all food and beverage purchases for household consumption, including items purchased for meals prepared at home. The formula used was:

$$\begin{array}{c} \text{Proportion of Income Spent}\;\mathrm{on}\;\text{Food}\;\left(\%\right)\\ =\left(\text{Total Monthly Food Expenditure}/\text{Total Monthly Household Income}\right)\\ \times 100. \end{array}$$

#### 2.3.3 Malnutrition Universal Screening Tool (MUST)

The Malnutrition Universal Screening Tool (MUST) is a useful tool for population‐wide screening and rapid assessment of malnutrition risk (Elizabeth Weekes et al. 2004). It is a five‐step screening tool designed to identify adults who are malnourished or at risk of malnutrition (Supporting Information S1). The tool assesses malnutrition risk using three independent criteria: current BMI, unintentional weight loss, and the presence of an acute disease effect that has caused or is likely to cause no nutritional intake for more than 5 days. Each parameter is scored as 0, 1, or 2. The overall risk of malnutrition is then categorized as low (score = 0), medium (score = 1), or high (score ≥ 2). Each of these criteria can independently predict clinical outcomes depending on the clinical context, but their combined use provides stronger predictive value than when used individually (Elia 2003; Stratton et al. 2004).

#### 2.3.4 Household Food Insecurity Access Scale (HFIAS)

The Household Food Insecurity Access Scale (HFIAS) is a survey instrument used to assess household food security status. It is an internationally recognized tool developed by the Food and Nutrition Technical Assistance (FANTA) project under the United States Agency for International Development (USAID) to estimate the prevalence of food insecurity (Coates et al. 2007; Deitchler et al. 2011). The scale aims to evaluate household experiences related to food insufficiency, lack of dietary diversity, food scarcity, and sacrifices made to obtain food. The HFIAS consists of nine items that assess whether households experienced specific conditions of food insecurity during the past 30 days (Supporting Information S2). These items reflect various degrees and dimensions of food insecurity, and include the following experiences:

- Worrying about food availability
- Inability to eat preferred foods
- Eating only a few kinds of foods
- Eating foods they really did not want to eat
- Eating smaller meals than needed
- Eating fewer meals per day
- Having no food at all in the household
- Going to sleep hungry
- Going a whole day and night without eating

Each question is asked with a 30‐day recall period. For each item, participants are first asked an *occurrence* question—whether the experience occurred at all in the past 4 weeks (yes or no). If the answer is “yes,” a *frequency‐of‐occurrence* question is then asked to determine how often the experience occurred. Responses are classified as:

- Rarely (once or twice),
- Sometimes (3 to 10 times),
- Often (more than 10 times).

The HFIAS questions are organized into three conceptual domains of food insecurity as defined by Deitchler et al. (2011):

- Anxiety and uncertainty about household food supply (1 item),
- Insufficient food quality (3 items), and
- Insufficient food intake and its physical consequences (5 items).

These domains help capture the multidimensional nature of food insecurity related to access and allow for the categorization of food security status into various levels of severity.

##### 2.3.4.1 Calculation and Categorization of HFIAS Scores

When used as a continuous indicator, each of the nine HFIAS questions is scored from 0 to 3, with 3 indicating the highest frequency of occurrence. The total HFIAS score ranges from 0 to 27. Higher scores reflect more severe experiences of food insecurity, while lower scores indicate less exposure to food insecurity.

Household food insecurity status was categorized into four levels based on clustering of participant responses:

- **Food‐secure household**: No experience of food insecurity‐related conditions. There may be rare concerns about food access.
- **Mildly food‐insecure household**: Occasional or frequent concerns about food sufficiency and/or rare inability to consume preferred foods, monotonous diets, or undesirable foods. However, the household does not reduce food quantity or experience the three most severe conditions: no food in the household, going to bed hungry, or going a whole day and night without eating.
- **Moderately food‐insecure household**: Household frequently consumes low‐quality diets; may sometimes or often eat monotonous or undesirable foods. Additionally, there may be rare or occasional reductions in meal size or frequency. However, the most severe three conditions are not observed.
- **Severely food‐insecure household**: Household frequently reduces meal quantity and/or even rarely experiences the three most severe conditions. The presence of even one of these conditions (e.g., not eating for an entire day and night) within the past month indicates severe food insecurity (Coates et al. 2007).

For the purpose of this study, food‐secure individuals and those with mild food insecurity were combined into a single category. This decision was based on two considerations: (1) previous studies have shown that mild food insecurity, characterized primarily by anxiety about food access and minor reductions in dietary quality without significant reductions in food quantity, may not yet be associated with measurable differences in anthropometric outcomes such as BMI (Franklin et al. 2012; Moradi et al. 2019); and (2) the primary focus of this study was to examine the associations between moderate‐to‐severe food insecurity and obesity, as these levels are more likely to be associated with clinically meaningful dietary changes and weight‐related outcomes (Coates et al. 2007; Deitchler et al. 2011). This approach is consistent with previous studies that have similarly combined food‐secure and mildly food‐insecure groups for analyzes focusing on obesity and dietary outcomes (Jung and Park 2025b; Lee et al. 2024).

#### 2.3.5 Assessment of Food Consumption Frequency and Quantity

##### 2.3.5.1 Frequency

Short‐term dietary records (covering only a few days) are insufficient to reflect overall dietary patterns, as they fail to capture day‐to‐day and seasonal variations in food intake (Cade et al. 2002; Willett 2013). While maintaining continuous dietary records over the long term is difficult, costly, and may alter participants' eating behaviors (Thompson et al. 2018), FFQs have been widely validated as a practical and cost‐effective method for assessing usual dietary intake over extended periods (Biro et al. 2002; Willett 2013). Although the FFQ used in this study assessed intake over the past month, it has been shown that well‐designed FFQs can reliably capture habitual dietary patterns, particularly when the recall period is limited to 1 month, as this minimizes recall bias while still reflecting typical consumption (Prentice et al. 2011; Subar et al. 2015). The FFQ used in this study was adapted from the validated food frequency questionnaire developed by Rakicioglu et al. (2025) and was further confirmed using the BeBiS (Nutrition Information System) software (2021).

##### 2.3.5.2 Quantity

For each food item, the portion size consumed in a single intake was recorded using common household measures (e.g., cup, plate, spoon). The weight in grams corresponding to each measure was determined using the Food and Meal Photograph Catalogue (Rakicioglu et al. 2025). These values were also confirmed through the BeBiS (Nutrition Information System) software (Ebis Ltd. 2021). Using frequency and single‐serving quantities, the average daily intake of each food was calculated. For example, a food item consumed every meal was multiplied by 3, whereas a food consumed once a month was multiplied by 0.033 to estimate its daily average intake (Prentice et al. 2011).

Food group unavailability was assessed by asking participants whether they had consumed each food group in the past year. The food groups assessed for unavailability included dairy products, meat‐egg‐legumes, vegetables‐fruits, cereals, and snack foods. Daily food intake (in grams/day) was assessed for dairy products, meat‐egg‐legumes, vegetables‐fruits, and cereals. Snack foods were not included in the daily intake analysis because the FFQ assessed only the frequency of snack consumption, not the quantity (Supporting Information S3).

### 2.4 Statistical Analysis

Data were analyzed using IBM SPSS Statistics (Version 20.0; IBM Corp. 2011) and Jamovi (Version 2.6; Jamovi Project 2025) software. The Kolmogorov–Smirnov test was used to assess the normality of data distribution. Parametric tests were applied to normally distributed data, while non‐parametric tests were used for non‐normally distributed data. The following statistical methods were employed: Kruskal–Wallis test, Pearson's chi‐square test, binary logistic regression, multinomial logistic regression, and moderation analysis. Covariates included in the regression models were selected a priori based on existing literature demonstrating associations with food insecurity and/or obesity. Only basic demographic factors consistently identified as key determinants were included: age, sex, education level, residence (rural/urban), poverty status, social support, and total number of employed household members. Moderation analysis was conducted to examine whether food insecurity (measured by HFIAS scores) moderates the relationship between daily intake of major food groups (dairy products, meat‐egg‐legumes, vegetables‐fruits, and cereals) and BMI. The moderation model was tested using the PROCESS macro for SPSS. Simple slope analysis was performed to examine the conditional effects of food group intake on BMI at low (−1 SD), average (mean), and high (+1 SD) levels of food insecurity. Mediation analysis was not performed due to the cross‐sectional nature of the data, which precludes the temporal ordering required for causal mediation. A *p*‐value of less than 0.05 was considered statistically significant.

## 3 Results

The mean age of the participants included in the study was 34.70 ± 13.84 years (range, 18–85). Of the participants, 389 (45.3%) were male and 470 (54.7%) were female. The prevalence of severe food insecurity among participants was 12.3%, while moderate food insecurity affected 9.7% of participants. When sociodemographic characteristics were evaluated according to food insecurity subgroups, the risk of severe food insecurity was found to prevail among males, individuals residing in rural areas, and those with incomes below the poverty threshold. Furthermore, participants with severe food insecurity had higher median BMI values, lower per capita income, and were less likely to receive social support for food needs. Among individuals with moderate or severe food insecurity, the percentage of monthly income spent on food was significantly higher, and the frequency of meal reduction was significantly higher compared to those who were food secure or mildly food insecure (*p* < 0.05; Table 1).

**TABLE 1:** Comparison of sociodemographic variables by food‐insecurity groups.

Characteristics	Food‐insecurity groups *n* (%) or median [IQR]			*p*
	No insecurity + Mild insecurity (*n* = 670)	Moderate insecurity (*n* = 83)	Severe insecurity (*n* = 106)	
Age	29 [20]	33 [23]	34 [27]	0.032*
Body mass index	24.6 [5.7]	24.6 [5.7]	25.9 [6]	0.002*
Sex				
Male	297 (44.3)	33 (39.8)	59 (55.7)	0.027*
Female	373 (55.7)	50 (60.2)	47 (44.3)	
Educational status				
High school or below	527 (78.7)	71 (85.5)	86 (81.1)	0.312
University or higher	143 (21.3)	12 (14.5)	20 (18.9)	
Marital status				0.309
Married	288 (43)	43 (51.8)	46 (43.4)	
Unmarried	382 (57)	40 (48.2)	60 (56.6)	
Employment status				
Employed	383 (57.2)	40 (48.2)	53 (50)	0.142
Unemployed	255 (38.1)	39 (47)	43 (40)	
Retired	32 (4.8)	4 (4.8)	10 (9.4)	
Residence				
Rural	260 (38.8)	41 (49.4)	53 (50)	0.026*
Urban	410 (61.2)	42 (50.6)	53 (50)	
Household income				
≥Poverty threshold	637 (95.1)	70 (84.3)	92 (86.8)	< 0.001*
<Poverty threshold	33 (4.9)	13 (15.7)	14 (13.2)	
Proportion of income spent on food (%)	25 [20]	30 [20]	32 [20]	< 0.001*
Per‐capita income (United States Dollars)	19.9 [17.02]	13.9 [7.8]	13.4 [10.1]	< 0.001*
Receiving social support for food needs				
Yes	166 (24.8)	27 (32.5)	18 (17)	0.046*
No	504 (75.5)	56 (67.5)	88 (83)	
Change in number of meals (past year)				
No change	530 (79.1)	53 (63.9)	67 (63.2)	< 0.001*
Decreased	88 (13.1)	25 (30.1)	31 (29.2)	
Increased	52 (7.8)	5 (6)	8 (7.5)	

* Statistically significant.

Among groups with moderate and severe food insecurity, obesity rates were found to be significantly higher, whereas there was no significant difference in malnutrition rates. When food groups that were *not consumed* in the past year were examined according to the levels of food insecurity (i.e., the proportion of participants who reported not consuming each food group) it was observed that the inability to access foods such as “meat‐egg‐legumes,” dairy products, fruits and vegetables, as well as oils and sugars, was significantly more common among individuals with moderate and severe food insecurity. The rate of not being able to purchase grain group foods (cereals) in the last year was found to be significantly higher in those with severe food insecurity compared to those with moderate–mild or no food insecurity. It was also observed that individuals with moderate or severe food insecurity consumed lower daily amounts of dairy products and food from the “meat‐egg‐legume” group, while cereal consumption was significantly higher (Table 2).

**TABLE 2:** Comparison of nutrition‐related characteristics according to food insecurity groups.

	Food insecurity groups *n* (%) or median [IQR]			*p*
	No insecurity + mild insecurity (*n* = 670)	Moderate insecurity (*n* = 83)	Severe insecurity (*n* = 106)	
Food unavailability in the past year				
Present	235 (35.1)	72 (86.7)	87 (82.1)	< 0.001*
Absent	435 (64.9)	11 (13.3)	19 (17.9)	
Malnutrition risk				
Low risk	604 (94.4%)	67 (91.8%)	87 (92.6%)	0.452
Moderate risk	28 (4.4%)	5 (6.8%)	4 (4.3%)	
High risk	8 (1.2%)	1 (1.4%)	3 (3.2%)	
Obesity				
< 30	640 (95.5)	73 (88.0)	94 (88.7)	< 0.001
≥ 30	30 (4.5)	10 (12.0)	12 (11.3)	
Food groups				
Meat, eggs, fish, poultry	253 (37.8)	74 (89.2)	85 (80.2)	< 0.001*
Legumes	8 (1.12)	7 (8.4)	16 (15.1)	< 0.001*
Dairy products	40 (6)	24 (28.9)	35 (33)	< 0.001*
Vegetables and fruits	26 (3.9)	16 (19.3)	29 (27.4)	< 0.001*
Cereals	5 (0.7)	2 (2.4)	16 (15.1)	< 0.001*
Snack foods	36 (5.4)	9 (10.8)	16 (15.1)	< 0.001*
Daily food intake (g/day)				
Milk and dairy products (g/day)	267.31 [244.04]^a,b^	205.25 [199.17]^a^	175.82 [241.06]^b^	< 0.001*
Meat, eggs, legumes (g/day)	104.99 [97.49]^a,b^	99.7 [60.26]^a^	68.52 [67.07]^b^	< 0.001*
Vegetables and fruits (g/day)	251.17 [187.45]	225.31 [134.45]	241.37 [196.02]	0.175
Cereals (g/day)	152.24 [138.87]	148.1 [113.58]	186.19 [201.61]	0.029*

*Note:* Food group unavailability represents the proportion of participants who reported not consuming each food group in the past year. Daily food intake (g/day) was assessed for dairy products, meat‐egg‐legumes, vegetables‐fruits, and cereals. Snack foods were not included in the daily intake analysis as the FFQ assessed only frequency of consumption. ^a^ or ^b^ Post hoc analysis results with different letter designations indicating statistically different groups.

* Statistically significant.

The multinomial logistic regression analysis model examining factors associated with moderate and severe food insecurity was found to be statistically significant (Model Fitting Information, *p* < 0.001), with good model fit (Pearson goodness‐of‐fit test, *p* = 0.271) and a Nagelkerke pseudo *R* ^2^ value of 0.097. Compared to the “no or mild food insecurity” group, the odds of moderate food insecurity were 2.13 times higher among those living in rural areas, while they were 4.16 times lower in households with income at or above the poverty line (OR = 0.249). The odds of severe food insecurity were 1.59 times higher among males and 2.50 times higher among those living in rural areas. Each one‐unit increase in age was associated with a 1.027‐fold increase in the odds of severe food insecurity, which were 2.8 times lower in households with income at or above the poverty line (OR = 0.356), and 1.87 times lower among individuals receiving social support (OR = 0.532; Table 3).

**TABLE 3:** Factors associated with food insecurity: Multinomial logistic regression analysis.

Food insecurity groups		*B*	*p*	OR	95% CI for OR	
					Lower bound	Upper bound
Modarate	Intercept	−2.628	< 0.001			
	Age	0.019	0.061	1.019	0.999	1.039
	Total number of household members	0.055	0.477	1.056	0.908	1.229
	Sex = Male	−0.141	0.562	0.869	0.540	1.398
	Sex = Female	0	—	—	—	—
	Poverty status = No	−1.391	< 0.001*	0.249	0.120	0.515
	Poverty status = Yes	0	—	—	—	—
	Education level = University	0.729	0.069	2.074	0.946	4.545
	Education level = High school or below	0	—	—	—	—
	Marital status = Married	−0.037	0.899	0.964	0.547	1.697
	Marital status = Other	0	—	—	—	—
	Social support = Yes	0.244	0.379	1.276	0.741	2.198
	Social support = No	0	—	—	—	—
	Residence = Rural	0.759	0.010*	2.135	1.199	3.804
	Residence = Urban	0	—	—	—	—
Severe	Intercept	−3.052	< 0.001			
	Age	0.026	0.005*	1.027	1.008	1.046
	Total number of household members	0.109	0.096	1.115	0.981	1.266
	Sex = Male	0.466	0.032*	1.593	1.041	2.438
	Sex = Female	0	—	—	—	—
	Poverty = No	−1.034	0.004*	0.356	0.177	0.716
	Poverty = Yes	0	—	—	—	—
	Education level = University	0.636	0.067	1.889	0.956	3.730
	Education level = High school or below	0	—	—	—	—
	Marital status = Married	−0.483	0.077	0.617	0.361	1.054
	Marital status = Other	0	—	—	—	—
	Social support = Yes	−0.631	0.035*	0.532	0.296	0.955
	Social support = No	0	—	—	—	—
	Residence = Rural	0.918	0.001*	2.505	1.459	4.300
	Residence = Urban	0	—	—	—	—

Abbreviations: CI, confidence interval; OR, odds ratio; ref, reference group.

* Statistically significant.

The logistic regression model constructed to predict obesity was found to be statistically significant (Omnibus test, *p* < 0.001). The model demonstrated an adequate fit (Nagelkerke *R* ^2^ = 0.154) and an overall classification accuracy of 85.3%. The dependent variable of the model was obesity (BMI ≥ 30). Among the independent variables included in the model, severe food insecurity was associated with a 2.075‐fold increase in the odds of obesity, and each one‐unit increase in age was associated with a 1.045‐fold increase in the odds of obesity. The odds of obesity were 2.55 times lower among individuals with a high school education or less (OR = 0.392), and 1.91 times lower among those receiving social support (OR = 0.522) (Table 4).

**TABLE 4:** Factors associated with obesity: Binary logistic regression analysis.

	B	*p*	OR	95% CI for OR	
				Lower	Upper
Food insecurity (ref: none + mild)		0.055			
Food insecurity (moderate)	0.352	0.316	1.422	0.715	2.829
Food insecurity (severe)	0.730	0.017*	2.075	1.140	3.779
Age	0.044	< 0.001*	1.045	1.031	1.060
Sex (ref: Male)	0.082	0.693	1.085	0.723	1.629
Residence (ref: Rural)	−0.117	0.641	0.890	0.545	1.454
Poverty (ref: No)	0.162	0.689	1.176	0.532	2.601
Education (ref: University)	−0.937	0.024*	0.392	0.173	0886
Total number of employed household members	0.133	0.088	1.142	0.980	1.331
Social support (ref: No)	−0.650	0.006*	0.522	0.330	0.827
Presence of food unavailability (ref: Yes)	0.273	0.259	1.313	0.818	2.108
Constant	−3.611	< 0.001	0.027		

Abbreviations: CI, confidence interval; OR, odds ratio; ref, reference group.

* Statistically significant.

**FIGURE 1:**
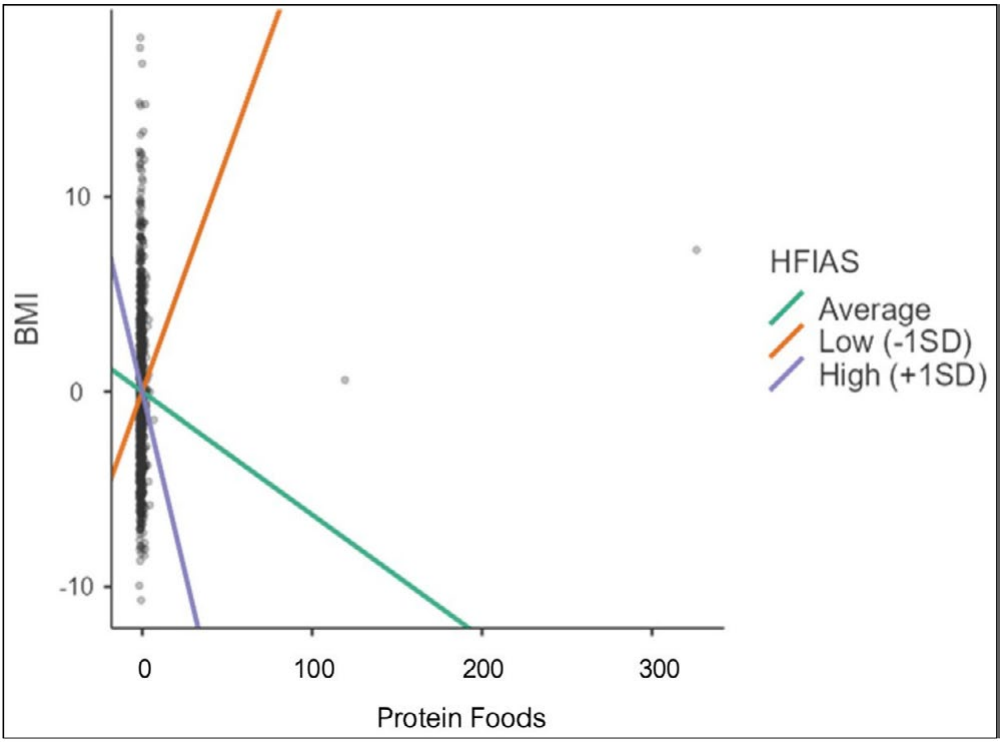
Moderating role of food insecurity in the relationship between the average daily intake of protein‐group foods and body mass index (simple slope plot): protein foods, foods rich in protein like meat, poultry, fish, legumes (in grams/day); BMI, body mass index; HFIAS, Household Food Insecurity Access Scale scores; SD, standard deviation.

Moderation analysis revealed that food insecurity significantly moderated the relationship between protein‐rich food intake and BMI (interaction *p* = 0.003). Simple slope analysis showed that the direction and strength of this association differed across levels of food insecurity. At lower levels of food insecurity (−1 SD), protein‐rich food intake was positively associated with BMI (β = 0.00242, *p* < 0.05), whereas at higher levels of food insecurity (+1 SD), this association reversed direction and became negative (β = −0.00368, *p* < 0.05). No significant moderation effects were observed for dairy products, vegetables and fruits, or cereals (Table 5, Figure 1).

**TABLE 5:** Moderation analysis of food insecurity (HFIAS score) on the association between daily food group intake and BMI.

Food group (g/day)	Moderator	Dependent variable	Interaction *p*	β (average)	β at low FI (−1 SD)	β at high FI (+1 SD)
Dairy products	HFIAS score	BMI	0.153	0.00371	0.00313	0.00710
Meat, poultry, fish, legumes	HFIAS score	BMI	0.003*	−0.00063	0.00242*	−0.00368*
Vegetables and fruits	HFIAS score	BMI	0.235	−0.00364	0.00497	−0.00120
Cereal‐based foods	HFIAS score	BMI	0.157	0.00190	0.00010	0.00270

Abbreviations: BMI, body mass index; g, gram (s); HFIAS, Household Food Insecurity Access Scale score; ref, reference group; SD, standard deviation.

* Statistically significant.

## 4 Discussion

Turkey has experienced a period of sustained food price inflation since 2020, driven by various economic and environmental factors (AFAD 2023; FAO 2023; TUIK 2023; World Bank 2023). While our study did not directly assess the specific contributions of these factors, the contextual backdrop of rising food prices informed our investigation of food insecurity and its relationship with obesity. According to the results of our study, the prevalence of general food insecurity among individuals aged 18 or older is 69.8%, mild food insecurity is 52.6%, moderate food insecurity is 9.7%, and severe food insecurity is 12.3%. According to the FAO (2022) report, the proportion of undernourished people in Turkey was below 2.5% between 2019 and 2021. However, our findings indicate that this proportion has increased over the last 5 years, raising concern. Recent studies conducted in Turkey have similarly reported increasing rates of food insecurity across various populations, including refugees, adults, children, the elderly, women of reproductive age, and households with children. These studies consistently identified declining income as a significant risk factor for food insecurity. Consistent with these findings, our study also found that having an income above the poverty line was protective against food insecurity (Aytekin Sahin and Mengi Celik 2024; Bulucu Büyüksoy et al. 2022; Esin et al. 2024; Simsek et al. 2013; Tari Selcuk et al. 2023).

A meta‐analysis examining the relationship between food insecurity and adverse health outcomes in adults found a direct link between food insecurity and 22 adverse health outcomes in adults that included risk of being overweight, binge eating, stress, anemia, long or short sleep duration, suicide attempts, obesity, diabetes, hypertension, anxiety, multiple morbidity, depression, poor sleep quality, suicidal thoughts, opportunistic infections, low CD4 count, excessive or inadequate weight gain during pregnancy, and pre‐pregnancy overweight or obesity. A significant portion of these adverse health outcomes was reported as directly or indirectly related to obesity (Moosavian et al. 2026).

There are findings indicating that, in addition to obesity's direct effect on the emergence of health problems related to food insecurity, its mediator and moderator roles are also significant (Leung et al. 2025). These findings suggested that the relationship between food insecurity and obesity was more complex than previously thought. In our study, the impact of food insecurity on obesity was evaluated. Obesity prevalence was found to be higher among individuals experiencing moderate and severe food insecurity. Severe food insecurity was associated with a 2.075‐fold increase in obesity risk. Food insecurity was also identified as a significant moderator in the relationship between protein‐rich food consumption and BMI, with the association weakening at higher levels of food insecurity. Individuals with moderate or severe food insecurity had lower daily intake of protein‐rich foods and higher consumption of cereals and snacks. Those with moderate and severe food insecurity had lower proportions of protein foods consumed daily and higher proportions of grain foods and snacks. The risk of food insecurity was higher among individuals living in rural areas, those with lower income, males, older adults, and those not receiving food assistance.

In a study conducted in Korea by Jung and Park, the prevalence of food insecurity was found to be 4%. Among women, a positive association was reported between food insecurity and obesity (OR = 1.37; 95% CI [1.03–1.81]). Subgroup analyzes based on sociodemographic factors indicated a positive association between food insecurity and obesity among older women and women living in urban areas (Jung and Park 2025b). In another study conducted among Korean adults, food insecurity was found to be negatively associated with overweight and mild obesity among men, but positively associated with obesity among women (Chun et al. 2015). Another study showed that among Korean adults aged 19 and older, food insecurity was associated with metabolically unhealthy obesity (Lee et al. 2024). A meta‐analysis also found that adults experiencing food insecurity were at higher risk for obesity (OR = 1.15). Subgroup analyzes by gender revealed that this risk was more pronounced in women compared to men (OR = 1.26). Analyzes based on the severity of food insecurity indicated that individuals experiencing severe food insecurity might have a higher risk of being underweight (49%) than being overweight (37%) or obese (29%). Moreover, subgroup analyzes demonstrated that in countries with lower levels of economic development, abnormalities related to body weight tended to manifest more frequently as undernutrition rather than obesity (Moradi et al. 2019). In a study by Poulsen et al. (2019), adolescents living in food‐insecure households were found to have higher average BMI scores and lower scores on healthy home food environment assessments.

In our study, severe food insecurity was found to increase the risk of obesity, and no significant difference was observed by sex. The classification of our country as an upper‐middle‐income economy might have contributed to weight‐related abnormalities manifesting more as overweight and obesity rather than undernutrition. Additionally, among individuals experiencing food insecurity, a shift toward carbohydrate‐based dietary patterns (e.g., high bread consumption) and increased intake of high‐energy, low‐nutrient foods such as snacks might have contributed to weight gain. The effects of the inflationary period experienced in the past 5 years in our country have only recently begun to emerge. If the economic crisis and rising food prices persist, the situation may shift in the future toward increased undernutrition. The absence of differences in malnutrition rates among participants, alongside significantly higher obesity rates among those with moderate and severe food insecurity, supports this de facto nutritional shift.

In high‐income countries, food insecurity has been linked to obesity. This paradoxical relationship has been explained through the “insurance hypothesis,” which suggests that humans have evolved adaptive mechanisms that favor fat storage as a buffer against unpredictable access to food. The evolutionary rationale underlying this hypothesis has been supported by experiments in animals; however, the exact physiological mechanisms remain unclear. While food insecurity may induce short‐term overeating (hyperphagia), direct evidence that it increases total energy intake is lacking. Animal studies have shown that exposure to unpredictable food access can lead to increased metabolizable energy and decreased energy expenditure, potentially supporting weight gain even in the absence of increased food intake (Bateson and Pepper 2023).

In an experimental study by Gil et al. (2025), food insecurity was shown to increase fat mass and reduce lean body mass in both male and female mice fed a standard diet. Among male mice, food insecurity also induced the upregulation of metabolic pathways associated with fat accumulation. These findings suggest that food insecurity may trigger metabolic adaptations that promote fat storage. Understanding this paradoxical relationship between food insecurity and adiposity is critical for developing targeted interventions to address the disproportionate prevalence of obesity among socioeconomically disadvantaged populations (Gil et al. 2025). Our study contributed valuable evidence in this context. Contrary to the insurance hypothesis, the findings of our observational study suggested that food insecurity in our setting might lead to a dietary shift toward high‐energy, low‐quality foods.

In a study by Ashe and Lapane (2018) women experiencing food insecurity were found to be 1.4 times more likely to be obese. Additionally, women with food insecurity were less likely to report having strong social support. However, in that study, the way social support was measured did not moderate the relationship between food insecurity and obesity. In our study, no significant relationship was found between sex and obesity. However, the risk of severe food insecurity was 1.5 times higher among men, and the risk of obesity was lower among individuals who reported having social support. Lack of social support was more prevalent among men, which might be related to gender roles; men might be less likely to seek help or report a need for support. A study examining the dynamics of food insecurity in Turkey found that the likelihood of food insecurity decreased significantly as the education level and income increased, and was 4% higher among men than among women (İpek 2022).

Food insecurity may lead to irregular dietary patterns, characterized by undernutrition and food deprivation during periods of limited resources, followed by compensatory overeating during periods of greater availability. This cycle contributes to increased body fat. In addition to this pattern, the widespread availability of high‐calorie, low‐cost foods commonly consumed by food‐insecure individuals further reinforces this relationship (Brown et al. 2019). These mechanisms may serve as short‐term survival strategies under food‐insecure conditions; however, they come at the potential cost of increased disease risk. Ultimately, exposure to food insecurity may result in increased body fat, poor health outcomes, and shorter life expectancy (Bateson and Pepper 2023). A relationship appears to exist between increasing levels of food insecurity and abnormal body weight; however, due to high heterogeneity among studies, results should be interpreted with caution (Rezaei et al. 2024). It is important to note that our analysis focused on moderation rather than mediation due to the cross‐sectional design of the study. While we observed that food insecurity moderates the relationship between protein intake and BMI, the cross‐sectional nature of the data precludes causal interpretation of this moderating effect. Future longitudinal studies are needed to establish the temporal order of these relationships and to test whether dietary intake mediates the food insecurity–obesity association (Maxwell and Cole 2007; MacKinnon et al. 2012).

Although our study focuses on the economic accessibility dimension, the issue of food insecurity highlights the fragility of the system in terms of food availability and access, as well as the sustainability of the environment that underpins this availability. Stakeholders must therefore address the food system as a whole. In this context, not only consumer‐focused indicators (price affordability and quality) but also the sustainability of inputs on the production side, farmers' access to these inputs, and the resilience of infrastructure should be considered. The impact of vulnerabilities in agricultural production on food security has become more apparent, particularly in conjunction with climate change, political instability, and disruptions in supply chains (FAO et al. 2025; The Economist Group 2022). Furthermore, the interconnected nature of the food system reveals that political and social barriers affect not only food access but also complementary areas such as trade liberalization and logistics infrastructure. These factors can exacerbate inequalities, leading to increased food costs and greater dependence on chronic food aid. Particularly in low‐ and middle‐income countries, these vulnerabilities are known to increase the burden on health outcomes such as obesity, malnutrition, and diabetes. In this context, considering not only economic access but also availability, utilization, and stability dimensions in food security research will contribute to a more comprehensive understanding of health issues such as obesity (FAO et al. 2025; The Economist Group 2022).

In addition to its association with obesity, food insecurity has been increasingly recognized as a contributing factor to Metabolic Dysfunction‐Associated Steatotic Liver Disease (MASLD), the hepatic manifestation of obesity (Liu et al. 2026; Younossi et al. 2025; Zelber‐Sagi et al. 2024). The consumption of energy‐dense, low‐quality foods—often driven by financial constraints—can promote hepatic steatosis, insulin resistance, and inflammation (Krahmer et al. 2025; Jung and Park 2025a). Although the present study did not directly assess liver function or MASLD outcomes, the observed relationship between food insecurity and obesity suggests that food‐insecure individuals may also be at heightened risk for MASLD. Future research should incorporate biomarkers of liver health to further explore these pathways.

### 4.1 Limitations and Strengths

Several limitations should be considered when interpreting the findings of this study. First, the cross‐sectional design precludes establishing causal relationships or temporal ordering among food insecurity, dietary intake, and obesity. Therefore, our findings should be interpreted as associations rather than causal effects. Second, the reliance on self‐reported data for dietary intake, food expenditure, and income may have introduced information bias, as participants may have over‐ or under‐reported these variables. Third, the indirect assessment of obesity through BMI has well‐documented limitations, as BMI does not distinguish between fat mass and lean mass, nor does it capture fat distribution or the presence of obesity‐related metabolic complications. Recent diagnostic frameworks proposed by the Lancet Diabetes & Endocrinology Commission and the European Association for the Study of Obesity (EASO) have shifted toward a multidimensional clinical disease model that emphasizes excess adiposity, fat distribution, and obesity‐related organ dysfunction rather than BMI alone (Rubino et al. 2025; Di Vincenzo et al. 2026). The use of BMI‐based definitions in the present study may have resulted in misclassification and should be considered when interpreting the findings. Future studies should incorporate more comprehensive anthropometric measures such as waist circumference, waist‐to‐hip ratio, or direct adiposity assessments. Fourth, the use of a non‐probability sampling method (convenience sampling) may have resulted in selection bias, potentially limiting the representativeness of our sample and the generalizability of our findings to the broader Turkish adult population. Fifth, food insecurity was assessed solely through the economic access dimension using the HFIAS, without evaluating other important sub‐dimensions such as food availability, utilization, and stability. Future research should adopt more comprehensive food security frameworks that capture these multiple dimensions. Sixth, the FFQ used in this study assessed dietary intake over the past month and covered a limited number of broad food categories, which may have led to the loss of detailed nutritional information and potential misclassification of dietary exposures. Although the FFQ was adapted from a validated instrument and confirmed using BeBiS software (Rakicioglu et al. 2025; Ebis Ltd. 2021), the use of broad food groups may have masked more specific dietary patterns associated with food insecurity.

Seventh, the relatively low Nagelkerke *R* ^2^ values observed in our models (0.097 for the multinomial model and 0.154 for the obesity model) indicate that a considerable proportion of the variance in food insecurity and obesity remains unexplained. This suggests that the predictors included in our models—while statistically significant—are not sufficient to fully capture the complexity of these outcomes. Food insecurity and obesity are multifactorial phenomena influenced by a broad range of individual, social, environmental, and policy‐related factors. Unmeasured or imperfectly measured factors, such as physical activity, stress, depression, health literacy, and food environment characteristics, may have influenced the observed associations (residual confounding). Finally, the study population was drawn from primary care settings in Adana, Turkey, which may limit the generalizability of our findings to other regions or populations in Turkey. Despite these limitations, the study has several strengths. It is one of the few observational studies to examine the relationship between food insecurity and obesity in the general Turkish adult population, and it was conducted during a period of rising food prices, providing timely and relevant insights into this important public health issue. The use of a validated HFIAS alongside standardized anthropometric measurements also strengthens the reliability of our findings.

## 5 Conclusion

According to the findings of our study, severe food insecurity was associated with significantly higher odds of obesity. Food insecurity was associated with lower dietary protein intake and higher carbohydrate intake. In particular, individuals with moderate to severe food insecurity consumed more carbohydrates and snack foods. Our findings suggest that food insecurity is associated with obesity, and this association may be linked to dietary patterns characterized by lower protein intake and higher consumption of cereals and snack foods. These findings highlight the need for further research to further explore the factors underlying this association. While our results suggest that food‐insecure populations may be a key target for further research and interventions, the cross‐sectional design of this study precludes causal inference. Longitudinal studies and intervention trials are needed to establish the direction of the association between food security and obesity. Nevertheless, our findings underscore the importance of considering food insecurity as a potential factor associated with obesity in public health strategies.

## Author Contributions

**Burak Mete:** conceptualization, writing – original draft, writing – review and editing, methodology, formal analysis, software, project administration, supervision. **Hatice Merve Sadıkoğlu:** conceptualization, methodology, project administration, writing – review and editing, data curation. **Hakan Demirhindi:** conceptualization, writing – review and editing. **Şeyma Nur Kutay Güler:** conceptualization, methodology, data curation. **Fatma İrem Dağlı:** conceptualization, methodology, data curation. **Ferdi Tanır:** conceptualization, writing – review and editing.

## Funding

This work was supported by Türkiye Bilimsel ve Teknolojik Araştırma Kurumu.

## Ethics Statement

Ethical approval was obtained from the Non‐Interventional Research Ethics Committee of the Faculty of Medicine, Çukurova University, Adana, Turkey (Date: 07.02.2025, Decision No: 152).

## Consent

Written informed consent was obtained from all subjects involved in the study.

## Conflicts of Interest

The authors declare no conflicts of interest.

## Supporting information


**Data S1:** Supporting Information.

**Data S2:** Supporting Information.

**Data S3:** Supporting Information.

## Data Availability

The data that support the findings of this study are available on request from the corresponding author. The data are not publicly available due to privacy or ethical restrictions.
