# Supplementary material for: The Direct and Moderating Effect of Food Insecurity on Obesity—A Cross‐Sectional Study
Source: Food Sci Nutr. 2026 Jul 30;14(8):e72193. doi: 10.1002/fsn3.72193 (PMC13425597; doi:10.1002/fsn3.72193)
Supplement: Supplementary file 1 — Data S1: Supporting Information. [file FSN3-14-e72193-s003.docx]

**Malnutrition Universal Screening Tool (MUST)**

| **Step** | **Description** | **Details** | **Score** |
| --- | --- | --- | --- |
| **Step 1** | **BMI Score** | BMI (kg/m²):>30: Obese>20: Normal18.5–20: Low<18.5: Very low | >30 → 0  >20 → 0  18.5–20 → 1  <18.5 → 2 |
| **Step 2** | **Weight Loss Score** | Weight loss in the last 3–6 months (%):  <5  %5–10  %>10% | <5 → 0  5–10 → 1  >10 → 2 |
| **Step 3** | **Acute Disease Score** | Acute disease present or likelihood of no nutritional intake for >5 days | Yes → 2 |
| **Step 4** | **Total Malnutrition Risk** | Add up scores from Steps 1–3 |  |
| **Step 5** | **Nutrition Treatment and Management Guide** | Based on total score | Score 0: Low risk  Score 1: Medium risk  Score ≥2: High risk |
