# Supplementary material for: The Direct and Moderating Effect of Food Insecurity on Obesity—A Cross‐Sectional Study
Source: Food Sci Nutr. 2026 Jul 30;14(8):e72193. doi: 10.1002/fsn3.72193 (PMC13425597; doi:10.1002/fsn3.72193)
Supplement: Supplementary file 2 — Data S2: Supporting Information. [file FSN3-14-e72193-s001.docx]

**Household Food Insecurity Access Scale (HFIAS) Measurement Tool**

| No | Question | Response Question |
| --- | --- | --- |
| 1. | In the past four weeks, did you worry that your household would not have enough food? | 0 = No (skip to Q2)  1=Yes |
| 1.a | How often did this happen? | \| 1=Rarely (once or twice in the past four weeks)  2= Sometimes (three to ten times in the past four weeks)  3= Often (more than ten times in the past four weeks) \| \| \| \| --- \| --- \| --- \| \|  \|  \|  \|  \| \|  \| \| \| |
| 2. | In the past four weeks, were you or any household member not able to eat the kinds of foods you preferred because of a lack of resources? | 0 = No (skip to Q3)  1=Yes |
| 2.a | How often did this happen? | 1=Rarely (once or twice in the past four weeks)  2= Sometimes (three to ten times in the past four weeks)  3= Often (more than ten times in the past four weeks) |
| 3. | In the past four weeks, did you or any household member have to eat a limited variety of foods due to a lack of resources? | 0 = No (skip to Q4)  1 = Yes |
| 3.a | How often did this happen? | 1=Rarely (once or twice in the past four weeks)  2= Sometimes (three to ten times in the past four weeks)  3= Often (more than ten times in the past four weeks) |
| 4. | In the past four weeks, did you or any household member have to eat some foods that you really did not want to eat because of a lack of resources to obtain other types of food? | 0 = No (skip to Q5)  1 = Yes |
| 4.a | How often did this happen? | 1=Rarely (once or twice in the past four weeks)  2= Sometimes (three to ten times in the past four weeks)  3= Often (more than ten times in the past four weeks) |
| 5. | In the past four weeks, did you or any household member have to eat a smaller meal than you felt you needed because there was not enough food? | 0 = No (skip to Q6)  1 = Yes |
| 5.a | How often did this happen? | 1=Rarely (once or twice in the past four weeks)  2= Sometimes (three to ten times in the past four weeks)  3= Often (more than ten times in the past four weeks) |
| 6. | In the past four weeks, did you or any other household member have to eat fewer meals in a day because there was not enough food? | 0 = No (skip to Q7)  1 = Yes |
| 6.a | How often did this happen? | 1=Rarely (once or twice in the past four weeks)  2= Sometimes (three to ten times in the past four weeks)  3= Often (more than ten times in the past four weeks) |
| 7. | In the past four weeks, was there ever no food to eat of any kind in your household because of lack of resources to get food? | 0 = No (skip to Q8)  1 = Yes |
| 7.a | How often did this happen? | 1=Rarely (once or twice in the past four weeks)  2= Sometimes (three to ten times in the past four weeks)  3= Often (more than ten times in the past four weeks) |
| 8. | In the past four weeks, did you or any household member go to sleep at night hungry because there was not enough food? | 0 = No (skip to Q9)  1 = Yes |
| 8.a | How often did this happen? | 1=Rarely (once or twice in the past four weeks)  2= Sometimes (three to ten times in the past four weeks)  3= Often (more than ten times in the past four weeks) |
| 9. | In the past four weeks, did you or any household member go a whole day and night without eating anything because there was not enough food? | 0 = No (questionnaire is finished)  1 = Yes |
| 9.a | How often did this happen? | 1=Rarely (once or twice in the past four weeks)  2= Sometimes (three to ten times in the past four weeks)  3= Often (more than ten times in the past four weeks) |
