# Supplementary material for: The Direct and Moderating Effect of Food Insecurity on Obesity—A Cross‐Sectional Study
Source: Food Sci Nutr. 2026 Jul 30;14(8):e72193. doi: 10.1002/fsn3.72193 (PMC13425597; doi:10.1002/fsn3.72193)
Supplement: Supplementary file 3 — Data S3: Supporting Information. [file FSN3-14-e72193-s002.docx]

1. **Food Consumption Frequency Questionnaire**

|  | **Every meal** | **Every day** | **5-6 times per week** | **3-4 times per week** | **1-2 times per week** | **Once every 15 days** | **Once per month** | **Never** | **Portion** | **Weight/Volume(g/ml)** |
| --- | --- | --- | --- | --- | --- | --- | --- | --- | --- | --- |
| **Milk and dairy product** | | | | | | | | | | |
| Milk |  |  |  |  |  |  |  |  |  |  |
| Ayran(yoğurt-based drink) |  |  |  |  |  |  |  |  |  |  |
| Cheese |  |  |  |  |  |  |  |  |  |  |
| Yogurt |  |  |  |  |  |  |  |  |  |  |
| Ice cream |  |  |  |  |  |  |  |  |  |  |
| **Meat, eggs, legumes** | | | | | | | | | | |
| Red meat |  |  |  |  |  |  |  |  |  |  |
| Chicken |  |  |  |  |  |  |  |  |  |  |
| Fish |  |  |  |  |  |  |  |  |  |  |
| Eggs |  |  |  |  |  |  |  |  |  |  |
| Offal(e.g., liver) |  |  |  |  |  |  |  |  |  |  |
| Nuts and seeds |  |  |  |  |  |  |  |  |  |  |
| Legumes(e.g., chickpeas) |  |  |  |  |  |  |  |  |  |  |
| **Vegetables and fruits** | | | | | | | | | | |
| Leafy greens |  |  |  |  |  |  |  |  |  |  |
| Other vegetables |  |  |  |  |  |  |  |  |  |  |
| Potatoes |  |  |  |  |  |  |  |  |  |  |
| Fresh Fruits |  |  |  |  |  |  |  |  |  |  |
| Dried Fruits |  |  |  |  |  |  |  |  |  |  |
| **Cereals and grains** | | | | | | | | | | |
| White Bread |  |  |  |  |  |  |  |  |  |  |
| Whole wheat bread |  |  |  |  |  |  |  |  |  |  |
| Other breads |  |  |  |  |  |  |  |  |  |  |
| Rice |  |  |  |  |  |  |  |  |  |  |
| Bulgur(cracked wheat) |  |  |  |  |  |  |  |  |  |  |
| Pasta, noodles |  |  |  |  |  |  |  |  |  |  |
| Pastry(börek) |  |  |  |  |  |  |  |  |  |  |
| Cookies |  |  |  |  |  |  |  |  |  |  |
| Cake |  |  |  |  |  |  |  |  |  |  |
| Breakfast cereals |  |  |  |  |  |  |  |  |  |  |
| Popcorn |  |  |  |  |  |  |  |  |  |  |
| Chips,etc. |  |  |  |  |  |  |  |  |  |  |
| Other |  |  |  |  |  |  |  |  |  |  |
